# Residual coronary atherosclerotic risk and low LDL-cholesterol in chronic coronary syndromes

**DOI:** 10.1093/ehjimp/qyag021

**Published:** 2026-02-04

**Authors:** Danilo Neglia, Katia Pane, Mariaelena Occhipinti, Carmelo De Gori, Silvia Rocchiccioli, Rosetta Ragusa, Carlo Cavaliere, Mario Zanfardino, Concetta Prontera, Nicoletta Botto, Maria Sole Morelli, Antonio Morlando, Adrian Florentin Suman, Cecilia Vecoli, Emanuela Passaro, Luigi Coppola, Erica Maffei, Claudio Passino, Alberto Clemente, Filippo Cademartiri, Monica Franzese, Alessia Gimelli, Chiara Caselli

**Affiliations:** Cardio-Thoracic and Imaging Departments, Fondazione Toscana Gabriele Monasterio (FTGM), Via G. Moruzzi 1, Pisa 56124, Italy; Institute of Clinical Physiology, Consiglio Nazionale Delle Ricerche (CNR), Via G. Moruzzi 1, Pisa 56124, Italy; IRCCS SYNLAB SDN, Via Emanuele Gianturco 113, Napoli 80143, Italy; Cardio-Thoracic and Imaging Departments, Fondazione Toscana Gabriele Monasterio (FTGM), Via G. Moruzzi 1, Pisa 56124, Italy; Cardio-Thoracic and Imaging Departments, Fondazione Toscana Gabriele Monasterio (FTGM), Via G. Moruzzi 1, Pisa 56124, Italy; Institute of Clinical Physiology, Consiglio Nazionale Delle Ricerche (CNR), Via G. Moruzzi 1, Pisa 56124, Italy; Institute of Clinical Physiology, Consiglio Nazionale Delle Ricerche (CNR), Via G. Moruzzi 1, Pisa 56124, Italy; IRCCS SYNLAB SDN, Via Emanuele Gianturco 113, Napoli 80143, Italy; IRCCS SYNLAB SDN, Via Emanuele Gianturco 113, Napoli 80143, Italy; Cardio-Thoracic and Imaging Departments, Fondazione Toscana Gabriele Monasterio (FTGM), Via G. Moruzzi 1, Pisa 56124, Italy; Cardio-Thoracic and Imaging Departments, Fondazione Toscana Gabriele Monasterio (FTGM), Via G. Moruzzi 1, Pisa 56124, Italy; Cardio-Thoracic and Imaging Departments, Fondazione Toscana Gabriele Monasterio (FTGM), Via G. Moruzzi 1, Pisa 56124, Italy; Cardio-Thoracic and Imaging Departments, Fondazione Toscana Gabriele Monasterio (FTGM), Via G. Moruzzi 1, Pisa 56124, Italy; Institute of Clinical Physiology, Consiglio Nazionale Delle Ricerche (CNR), Via G. Moruzzi 1, Pisa 56124, Italy; Institute of Clinical Physiology, Consiglio Nazionale Delle Ricerche (CNR), Via G. Moruzzi 1, Pisa 56124, Italy; Cardio-Thoracic and Imaging Departments, Fondazione Toscana Gabriele Monasterio (FTGM), Via G. Moruzzi 1, Pisa 56124, Italy; Institute of Clinical Physiology, Consiglio Nazionale Delle Ricerche (CNR), Via G. Moruzzi 1, Pisa 56124, Italy; IRCCS SYNLAB SDN, Via Emanuele Gianturco 113, Napoli 80143, Italy; IRCCS SYNLAB SDN, Via Emanuele Gianturco 113, Napoli 80143, Italy; IRCCS SYNLAB SDN, Via Emanuele Gianturco 113, Napoli 80143, Italy; Cardio-Thoracic and Imaging Departments, Fondazione Toscana Gabriele Monasterio (FTGM), Via G. Moruzzi 1, Pisa 56124, Italy; Health Science Department, Scuola Superiore Sant’Anna, Piazza Martiri della Libertà 33, Pisa 56127, Italy; Cardio-Thoracic and Imaging Departments, Fondazione Toscana Gabriele Monasterio (FTGM), Via G. Moruzzi 1, Pisa 56124, Italy; IRCCS SYNLAB SDN, Via Emanuele Gianturco 113, Napoli 80143, Italy; IRCCS SYNLAB SDN, Via Emanuele Gianturco 113, Napoli 80143, Italy; Cardio-Thoracic and Imaging Departments, Fondazione Toscana Gabriele Monasterio (FTGM), Via G. Moruzzi 1, Pisa 56124, Italy; Cardio-Thoracic and Imaging Departments, Fondazione Toscana Gabriele Monasterio (FTGM), Via G. Moruzzi 1, Pisa 56124, Italy; Institute of Clinical Physiology, Consiglio Nazionale Delle Ricerche (CNR), Via G. Moruzzi 1, Pisa 56124, Italy

**Keywords:** chronic coronary syndromes, coronary atherosclerotic disease, cardiac computed tomography angiography, cardiometabolic risk, metabolic syndrome, LDL-cholesterol, diabetes

## Abstract

**Aims:**

Metabolic syndrome (Mes) and diabetes are emerging cardiometabolic determinants of coronary atherosclerotic disease (CAD) risk besides LDL-cholesterol (LDL-C) and established risk factors. We aimed to assess whether, in patients with chronic coronary syndrome (CCS), cardiometabolic risk is prevalent and independently associated with residual CAD risk in subjects with low LDL-C.

**Methods and results:**

The cross-sectional HURRICANE study (Health improvement by Understanding RR In CAd and NEw targets for treatment) included 479 patients with CCS (mean age 65 ± 11 years, 70% male), undergoing cardiac computed tomography angiography (CCTA). A severe/extensive CAD or a moderate-high CAD risk were defined, on patient level, by CCTA-derived CAD-RADS2 and Leiden scores. Metabolic syndrome was present in 31% of patients, diabetes or pre-diabetes in 21% and 26%, severe/extensive CAD and moderate-high Leiden score in 51% and in 61%, more frequently in the two lower LDL-C categories. Multivariate logistic regression models, included age, sex, smoking status, family history, LDL-C categories (<70, 70–99 100–129, and ≥130 mg/dL), MeS, or its components and medications. Independent predictors of moderate-high Leiden score were age, male sex, the lowest LDL-C category and MeS (OR 2.12, 95% CI: 1.26–3.56) or pre-diabetes (OR 1.90, 95% CI: 1.12–3.21) and diabetes (OR 6.13, 95% CI: 1.93–19.45). In the lowest LDL-C group, higher CAD-RADS2 and Leiden scores were more frequent in patients with cardiometabolic risk.

**Conclusion:**

Results of this cross-sectional study suggest that dysregulation of glucose metabolism is the prevalent component of residual cardiometabolic and coronary atherosclerotic risk in patients with CCS and low LDL-C under current treatment.

## Graphical Abstract

**Graphical Abstract:**
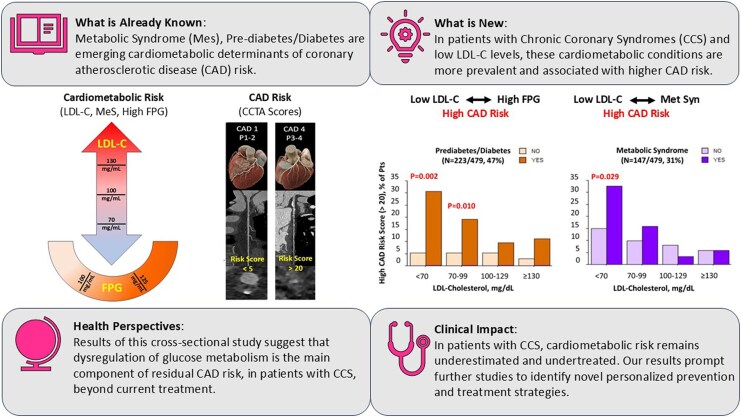

## Introduction

Coronary artery disease (CAD) remains the leading cause of cardiovascular morbidity and mortality worldwide.^1^ Despite improvements in pharmacological treatment, a substantial residual risk (RR) of CAD progression and events persists in patients with stable disease.^2^ RR is mainly attributed to a suboptimal control of LDL-cholesterol (LDL-C), systemic blood pressure, and thrombogenesis, and most efforts are focused on optimizing medical therapy.

Current guidelines recommend LDL-C targets < 70 mg/dL, < 55 mg/dL or even lower for patients with high, very high or extreme risk,^2–5^ based on evidence that very low LDL-C levels may reduce events without significant safety concerns.^6^

Nonetheless, some meta-analyses of statin trials^7^ as well as population-based studies, in patients with pre-existing ischaemic heart disease (IHD) taking statins^8–9^ or in subjects from the general population,^10,11^ yielded inconclusive results on the association between the magnitude of LDL-C reduction and outcomes.

Many factors might explain the RR of events in individuals with low LDL-C levels. Diabetes or pre-diabetes,^12,13^ elevated plasma levels of triglycerides (TGs)-rich lipoproteins and low HDL-cholesterol (HDL-C),^14,15^ central obesity and adipose tissue dysfunction, often accompanied by low-grade systemic inflammation,^16,17^ may cluster in the so-called metabolic syndrome (MeS)^18–21^ and are associated with atherogenesis and risk beyond LDL-C.^22^

Whether the prevalence of an adverse cardiometabolic profile in patients with low LDL-C levels might explain residual coronary atherosclerotic risk remains unclear. Mendelian randomization studies have shown that subjects with genetically determined low LDL-C levels have a higher prevalence of diabetes but lower CV events.^23^

The HURRICANE (‘Health Improvements by Understanding Residual Risk In CAd and NEw targets for prevention/treatment’) project aims to elucidate the major determinants of residual coronary atherosclerotic risk in patients with chronic coronary syndromes (CCS).^24^ It comprises a cross-sectional and a longitudinal observational studies performed in two separate cohorts of patients with CCS undergoing cardiac computed tomography angiography (CCTA). In this article, we present the findings of the cross-sectional study, focusing on the associations between lower LDL-C levels, adverse cardiometabolic/vascular risk profiles, and CCTA-derived, CAD severity/extent and risk.

## Methods

### Patients

The rationale and design of the cross-sectional HURRICANE study have been described previously.^24^ Among 947 patients undergoing a clinically indicated CCTA and enrolled in two independent prospective clinical trials, 561 fulfilled major HURRICANE inclusion and exclusion criteria and 479, with available complete information on risk factors and complete biochemical data, were included in the cross-sectional study (see Supplementary data online, *Table S1*). Eligible patients were adults aged 40–75 years, who provided written informed consent for the use of anonymized data in further analyses. Inclusion required availability of high quality, full accessible CCTA scans, availability of comprehensive demographic and clinical data, and biochemical markers of lipid and glucose metabolism, systemic inflammation, adipose tissue and renal function.

**Figure 1:**
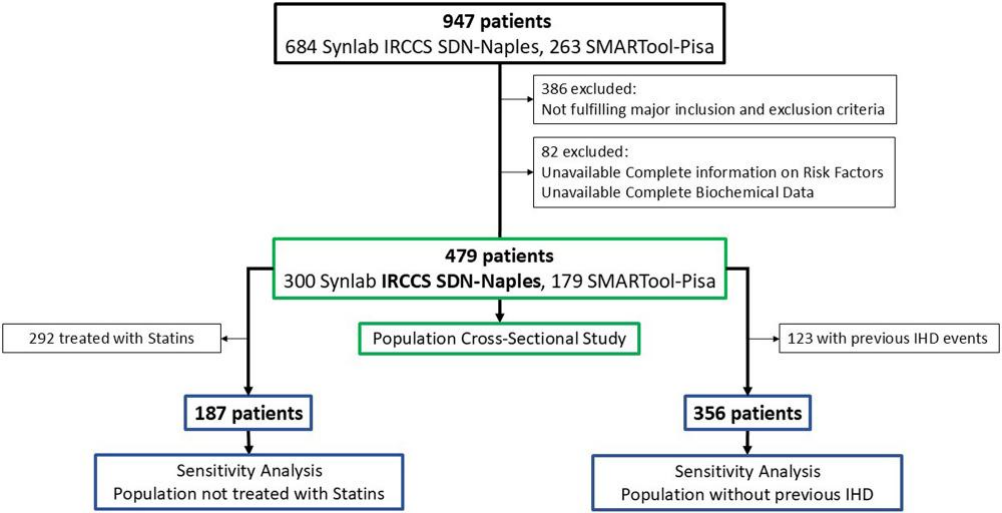
Patient flow-chart. Criteria for the selection of the present population of 479 patients, and the subpopulations of 356 patients without previous IHD events and of 187 patients not treated with statins for sensitivity analyses, from the initial 947 patients enrolled in two previous studies at Synlab IRCCS SDN-Naples or in Pisa and other European centres (participating to the SMARTool study) (Ref. ^24^). IHD, ischaemic heart disease; MI, myocardial infarction.

Among the 479 patients, 123 had pre-existing IHD requiring secondary prevention, including myocardial infarction (MI), percutaneous coronary intervention or coronary artery bypass grafting before the index CCTA. The remaining 356 patients without previous IHD events were analysed separately in sensitivity analyses (*Figure 1*).

### Clinical and biochemical characterization

Risk factor profile, laboratory data, and medication use were collected at the time of CCTA. Non cardiometabolic risk factors included age, sex, family history of cardiovascular disease (CVD), and smoking status. Cardiometabolic risk factors were defined according to international standards for cardiovascular risk assessment.^20,21,25,26^

To overcome the limitations of Friedewald or Martin equation in estimating LDL-C in patients with a low LDL-C level or hypertriglyceridaemia (TG > 400 mg/dL), LDL-C levels were calculated using the Sampson equation.^27^ Remnant-C was defined as total cholesterol minus LDL-C minus HDL-C, and non-HDL-C as total cholesterol minus HDL-C.

LDL-C levels were classified into four categories—low (<70 mg/dL), moderate (70–99.9 mg/dL), intermediate (100–129.9 mg/dL), and high (≥ 130 mg/dL)—according to guidelines-defined targets for primary and secondary prevention.^2–4,28,29^

Cardiometabolic risk factors composing the MeS, included: (i) hypertension under treatment; (ii) diabetes (treatment with hypoglycaemic agents or insulin or FPG ≥ 126 mg/dL) or pre-diabetes (FPG > 100 mg/dL); (iii) high body mass index (>30 kg/m2); (iv) low HDL-C (<40 mg/dL in males and <50 mg/dL in females); and (v) high TG (>150 mg/dL). The presence of MeS was defined by the combination of at least three of the five cardiometabolic risk factors in the individual patient.^18–21^ The homeostatic model assessment (HOMA) index was calculated and used as an index of insulin resistance and beta-cells function as well as a predictor of new-onset diabetes in non-diabetic patients.^30^

Cardiometabolic risk was also defined by categorical variables using the upper tertile (III tertile) of biochemical markers as follows: insulin resistance (HOMA Index >3.00), atherogenic dyslipidaemia (TG/HDL-C > 2.83 or remnant-C > 24 mg/dL), and low-grade systemic inflammation (IL-6 > 3.00 ng/L).

### CCTA analysis

CCTA scans were qualitatively and semi-quantitatively analysed according to CAD-reporting and data system (CAD-RADS) 2.0 classification.^31^ The degree of maximal coronary stenosis is represented by the CAD-RADS category (1–5), and the disease extent by the plaque burden category (P 1–4), as defined by the Segment Involvement Score (SIS). The CAD-RADS2 category ‘S/G’ identified patients previously revascularized with coronary stents (S) or bypass grafts (G). For each patient, the total number of segments with any plaque (SIS), the number of segments with non-calcified/mixed or calcified plaques was reported and the Leiden score^32^ was calculated as a synthetic index of CAD severity, extent, and associated risk.

Moderate-to-high CAD risk (Primary OUTCOME) was defined by a Leiden score ≥5.^32^ Severe and/or extensive CAD (Secondary OUTCOME) was defined as: CCTA-derived CAD-RADS2 score categories 3–5 and/or P3–4 and/or S/G.

### Statistical analysis

Continuous variables are expressed as mean ± standard deviation (SD) or as median with interquartile range (IQR) for skewed distributions. Categorical variables are expressed as absolute values (percentage). In patient groups stratified for LDL-C levels, continuous data were compared with the Mann–Whitney *U* test or Kruskal–Wallis test for unpaired data, whereas categorical variables were compared with χ^2^ test.

Multivariable logistic regression was used to estimate the association of single or combined risk factors with the outcomes. LDL-C categories were included in all models using intermediate range (100–129 mg/dL) as the reference. To control for potential confounders, two modelling strategies were applied: Model 1 adjusted for baseline covariates, including age (continuous variable), sex, smoking status, family history (CAD or CAD-related events) and MeS as a single composite variable; Model 2 included the same covariates. substituting MeS with cardiometabolic risk components. Both models were further adjusted for all medications assumed at the time of CCTA exam (each drug included as a dichotomous variable 0–1) (Models 1A and 2A). Covariate selection was based on clinical criteria, specifically focusing on established major CVD risk factors and relevant medication use. To confirm the robustness of the primary findings, sensitivity analyses were performed in patients not treated with statins and in patients without previous IHD events (*Figure 1*).

Potential interactions between LDL-C category, MeS category and pre-diabetes/diabetes category on the frequency of high Leiden CAD risk score (>20) were tested using F-statistics derived from model-based analysis of variance (ANOVA). ANOVA was also used to assess the potential interaction between LDL-C category and each biochemical cardiometabolic risk index (III tertile vs. I–II tertiles) on continuous values of Leiden CAD risk score and plaque burden defined by the number of segments with any plaque (SIS), non-calcified/mixed or calcified plaques. To confirm the robustness of the primary findings, sensitivity analyses were performed in patients without previous IHD events.

Statistical analyses were performed using SPSS software package (IBM SPSS Statistics, Version 26). All statistical tests were two-sided, with *P* < 0.05 deemed significant.

## Results

### Patients’ characteristics

The clinical characteristics, risk factors, medication use, and biochemical profiles are presented in the whole population and compared among patient groups defined according to LDL-C categories in *Table 1*.

**Table 1:** Baseline characteristics of the whole population and of patient groups defined according to LDL-cholesterol categories

Characteristics	ALL	LDL-cholesterol					LDL-cholesterol		
	*n* *=* *479*	Low < 70 mg/dL *n* = 110	Moderate 70–99.9 mg/dL *n* = 148	Intermediate 100–129.9 mg/dL *n* = 117	High ≥ 130 mg/dL *n* = 104	*P* value	Low-moderate < 100 mg/dL *n* = 258	Intermediate-high ≥ 100 mg/dL *n* = 221	*P* value
**Risk factors**									
Age, years	65 ± 11	67 ± 10 ^#^	66 ± 10 ^#^	64 ± 11	63 ± 10	0.027	67 ± 10	64 ± 11	0.004
Sex Male	334 (70)	88 (80)	102 (69)	79 (68)	65 (63)	0.039	190 (74)	144 (65)	0.044
Family history of CVD	266 (56)	57 (52)	82 (55)	73 (62)	54 (52)	0.335	139 (54)	127 (57)	0.431
Smoking	98 (20)	22 (20)	25 (17)	31 (26)	20 (19)	0.274	47 (18)	51 (23)	0.189
Hypertension	364 (76)	87 (79)	126 (85)	87 (74)	64 (62)	<0.001	213 (83)	151 (68)	<0.001
Diabetes	99 (21)	41 (37)	37 (25)	9 (8)	12 (12)	<0.001	78 (30	21 (10)	<0.001
Pre-diabetes	124 (26)	31 (28)	36 (24)	33 (28)	24 (23)		67 (26)	56 (26)	
Low HDL cholesterol	118 (25)	30 (27)	47 (32)	24 (21)	17 (16)	0.025	77 (30)	41 (19)	0.004
High triglycerides	123 (26)	25 (23)	41 (28)	32 (27)	25 (24)	0.768	66 (26)	57 (26)	0.958
High body mass index (>30)	100 (21)	26 (24)	35 (24)	20 (17)	19 (18)	0.451	61 (24)	39 (18)	0.108
Metabolic syndrome	147 (31)	43 (39)	57 (39)	30 (26)	17 (16)	<0.001	100 (39)	47 (21)	<0.001
**History and symptoms**									
Previous myocardial infarction	56 (12)	25 (23)	21 (14)	6 (5)	4 (4)	<0.001	46 (18)	10 (5)	<0.001
Previous elective PCI and/or CABG	67 (24)	36 (33)	23 (16)	7 (6)	1 (1)	<0.001	59 (23)	8 (4)	<0.001
Previous IHD events	123 (26)	61 (55)	44 (29)	13 (11)	5 (5)	<0.001	105 (41)	18 (8)	<0.001
Angina (typical or atypical)	77 (16)	11 (10)	23 (16)	24 (21)	19 (18)	0.163	34 (13)	43 (19)	0.062
**Medications**									
Beta-blockers	223 (47)	65 (59)	78 (53)	50 (43)	30 (29)	<0.001	143 (55)	80 (36)	<0.001
Calcium antagonists	99 (21)	17 (15)	29 (20)	33 (28)	20 (19)	0.106	46 (18)	53 (24)	0.097
RAS inhibitors	253 (53)	56 (51)	87 (59)	64 (55)	46 (44)	0.138	143 (55)	110 (50)	0.217
Nitrates	15 (3)	2 (2)	8 (5)	2 (2)	3 (3)	0.267	10 (4)	5 (2)	0.312
Diuretics	72 (15)	18 (16)	27 (18)	17 (15)	10 (10)	0.289	45 (17)	27 (12)	0.111
Anti-diabetics	60 (13)	27 (25)	24 (16)	5 (4)	4 (4)	<0.001	51 (20)	9 (4)	<0.001
Anti-platelets	251 (52)	76 (69)	93 (63)	47 (40)	35 (34)	<0.001	169 (66)	82 (37)	<0.001
Statins	292 (61)	86 (78)	111 (75)	54 (46)	41 (39)	<0.001	197 (76)	95 (43)	<0.001
**Biochemical markers**									
Total-cholesterol, mg/dL	174 ± 44	124 ± 18 ^††,^**^,##^	157 ± 17 **^,##^	189 ± 20 ^##^	233 ± 27	<0.001	143 ± 24	210 ± 32	<0.001
LDL-cholesterol, mg/dL	99 ± 38	54 ± 11 ^††,^ **^,##^	83 ± 9 **^,##^	113 ± 8 ^##^	155 ± 21	<0.001	71 ± 18	133 ± 26	<0.001
HDL-cholesterol, mg/dL	52 ± 15	48 ± 13 *^,##^	50 ± 15 ^##^	53 ± 18	55 ± 12	0.001	49 ± 14	54 ± 16	<0.001
Non-HDL-cholesterol, mg/dL	122 ± 40	75 ± 14 ^††,^**^,##^	107 ± 13 **^,##^	135 ± 15 ^##^	178 ± 24	<0.001	93 ± 21	156 ± 29	<0.001
Remnant-cholesterol, mg/dL	22.7 ± 11.4	21.8 ± 10.2	23.1 ± 10.7	22.5 ± 12.8	23.3 ± 11.9	0.551	22.6 ± 10.5	22.9 ± 12.4	0.611
Triglycerides, mg/dL	128 ± 71	123 ± 75	131 ± 70	126 ± 75	130 ± 63	0.376	128 ± 72	128 ± 69	0.893
Triglycerides/HDL-Cholesterol	2.8 ± 2.3	2.8 ± 2.6	2.9 ± 2.1	2.8 ± 2.8	2.5 ± 1.6	0.494	2.9 ± 2.3	2.7 ± 2.3	0.158
Fasting plasma glucose, mg/dL	103 ± 23	108 ± 23 **^,##^	107 ± 31 **^,##^	97 ± 15	97 ± 13	<0.001	108 ± 28	97 ± 14	<0.001
Insulin, mU/L	13 ± 18	18 ± 32 **^,##^	12 ± 12 *^,#^	10 ± 9	10 ± 8	<0.001	103 ± 159	70 ± 59	<0.001
HOMA index	3.3 ± 4.9	4.8 ± 8.1 ^†,^**^,##^	3.4 ± 4.5 *^,#^	2.6 ± 2.8	2.3 ± 1.9	<0.001	4.0 ± 6.3	2.5 ± 2.4	<0.001
Adiponectin, μg/mL	4.8 ± 3.1	4.3 ± 3.0 *	4.6 ± 3.3 *	5.3 ± 3.2	4.9 ± 2.6	0.036	4.5 ± 3.2	5.1 ± 2.9	0.005
Creatinine, mg/dL	0.93 ± 0.23	0.99 ± 0.27 ^†,^*^,##^	0.93 ± 0.23	0.92 ± 0.19	0.90 ± 0.20	0.047	0.95 ± 0.25	0.91 ± 0.20	0.085
Interleukin-6, ng/L	2.9 ± 2.7	3.4 ± 3.8	2.7 ± 2.1	2.7 ± 2.0	2.9 ± 2.9	0.446	3.0 ± 3.0	2.8 ± 2.4	0.519

Continuous variables are presented as mean ± standard deviation, and logarithmic transformed for comparison when necessary. Categorical variables are presented as absolute *N* and (%). *P* values <0.05 indicate statistically significant differences among the 4 groups. † = *P* < 0.05 vs. Moderate LDL-C group. *, ** = *P* < 0.05, *P* < 0.01 vs. intermediate LDL-C group. ^#,##^ = *P* < 0.05, *P* < 0.01 vs. High LDL-C group. CVD, cardiovascular disease; PCI and/or CABG, coronary revascularization by percutaneous coronary intervention or coronary artery bypass grafting (5 over 67 elective revascularizations); Previous IHD events, previous ischaemic heart disease events (myocardial infarction or elective coronary revascularization).

The mean age of the 479 patients (334 males, 70%) was 65 ± 11 years. A family history of CVD was present in 56% of patients, hypertension in 76%, diabetes or pre-diabetes in 47% with other risk factors in lower proportions. The combination of at least three cardiometabolic risk factors identifying the MeS was observed in 147 patients (31%). A previous IHD event had occurred in 123 patients (25%) including MI in 56 and elective coronary revascularization in 67. At the time of index CCTA, only 16% of patients had stable chest pain.

Statins, RAS inhibitors, anti-platelets and beta-blockers were the most frequent drugs used (61, 53, 52, and 47%, respectively), followed by calcium antagonists (21%), diuretics (15%), anti-diabetics (13%), and nitrates (3%).

Patients with low or moderate LDL-C, as compared with patients with intermediate or high LDL-C, were older and more frequently males. They had a higher frequency of hypertension, diabetes or pre-diabetes, low HDL-C, MeS, and previous IHD events. They received more frequently beta-blockers, anti-diabetics, anti-platelets, and statins.

Notably, among biochemical markers, patients in the two lower LDL-C groups had lower levels of HDL-C and adiponectin (a marker of adipocytes dysfunction) and higher levels of glucose, insulin, and HOMA index. Creatinine was significantly higher in the lowest LDL-C group.

### CCTA-derived CAD severity/extent and risk

The presence, severity and extent of CAD, as clinically classified by CAD-RADS 2.0 scores, the Leiden risk score, and the coronary plaque burden and type from CCTA analysis, are presented for the entire cohort and stratified by LDL-C levels in *Table 2*.

**Table 2:** CCTA-derived CAD severity/extent, CAD risk and coronary plaque burden in the whole population and in patient groups defined according to LDL cholesterol categories

Characteristics	ALL	LDL-cholesterol					LDL-cholesterol		
	*n* = 479	Low < 70 mg/dL *n* = 110	Moderate 70–99.9 mg/dL *n* = 148	Intermediate 100–129.9 mg/dL *n* = 117	High ≥ 130 mg/dL *n* = 104	*P value*	Low-moderate < 100 mg/dL *n* = 258	Intermediate-high ≥ 100 mg/dL *n* = 221	*P value*
**Presence/severity of CAD** **(CAD-RADS2 score)**									
Absent CAD (0)	107 (22)	7 (6)	27 (18)	42 (36)	31 (30)	<0.001	34 (13)	73 (33)	<0.001
Non-obstructive CAD (1-2)	185 (39)	32 (29)	51 (35)	50 (43)	52 (50)		83 (32)	102 (46)	
Obstructive CAD (3-4-5)	65 (14)	10 (9)	27 (18)	12 (10)	16 (15)		37 (14)	28 (13)	
Revascularized CAD (SG)	123 (26)	61 (55)	44 (29)	13 (11)	5 (5)		105 (41)	18 (8)	
**Extension of CAD** **(CAD-RADS2 P score)**									
Absent CAD (0)	107 (22)	7 (6)	27 (18)	42 (36)	31 (30)	<0.001	34 (13)	73 (33)	<0.001
Less extensive (1-2)	153 (32)	17 (15)	43 (29)	42 (36)	51 (49)		60 (23)	93 (42)	
More extensive (3-4)	97 (20)	25 (23)	35 (24)	20 (17)	17 (16)		60 (23)	37 (17)	
Revascularized CAD (SG)	123 (26)	61 (55)	44 (29)	13 (11)	5 (5)		105 (41)	18 (8)	
**Secondary OUTCOME**									
Obstructive and/or more extensive CAD and/or Revascularized	238 (50)	86 (78)	86 (58)	37 (32)	29 (28)	<0.001	172 (67)	66 (30)	<0.001
**CAD Risk (Leiden score)**									
Score (continuous)	9.0 ± 8.1	12.5 ± 8.7 ^†,^**^,##^	9.6 ± 7.9 **^,##^	7.0 ± 7.4	6.9 ± 7.0	<0.001	10.9 ± 8.4	6.9 ± 7.2	<0.001
High risk (score > 20)	56 (12)	24 (22)	18 (12)	8 (7)	6 (6)	<0.001	42 (16)	14 (6)	<0.001
**Primary OUTCOME**									
Moderate-high risk (Score ≥ 5)	293 (61)	84 (76)	101 (68)	55 (47)	53 (51)	<0.001	185 (72)	108 (49)	<0.001
**Plaque burden and type**									
Segment involvement score (SIS)	3.3 ± 3.1	4.6 ± 3.2 ^††,^**^,##^	3.6 ± 3.1 *^,##^	2.6 ± 3.0	2.4 ± 2.6	<0.001	4.0 ± 3.2	2.5 ± 2.8	<0.001
Non-calcified/mixed plaques (*N*)	1.5 ± 2.3	2.3 ± 2.7 ^††,^**^,##^	1.5 ± 2.0	1.2 ± 2.3	1.2 ± 1.9	<0.001	1.8 ± 2.4	1.2 ± 2.1	0.002
Calcified plaques (*N*)	1.8 ± 2.2	2.3 ± 2.4 **^,##^	2.1 ± 2.4 *^,##^	1.4 ± 2.0	1.2 ± 1.9	<0.001	2.2 ± 2.4	1.3 ± 1.9	<0.001

Continuous variables are presented as mean ± standard deviation and logarithmic transformed for comparison when necessary. Categorical variables are presented as absolute *N* and (%). *P* values <0.05 show statistically significant differences among the four groups. † = *P* < 0.05 vs. moderate LDL-C group. *, ** = *P* < 0.05, *P* < 0.01 vs. intermediate LDL-C group. ^#,##^ = *P* < 0.05, *P* < 0.01 vs. High LDL-C group. CAD, coronary artery disease; CAD-RADS2 scores, see Methods section for details; SG, stent or graft; OUTCOMEs, see also Methods section for details.

Patients with low-moderate LDL-C levels had a lower prevalence of absent, non-obstructive and less extensive CAD and a higher prevalence of more severe (CAD-RADS2 = 3-4-5 or SG) and/or extensive CAD (*P* 3-4) (Secondary OUTCOME).

Patients with low-moderate LDL-C levels had higher values of the Leiden CAD Risk score, and a higher prevalence of either high CAD risk category (Leiden score > 20) and moderate-to-high CAD risk category (Leiden score ≥ 5, Primary OUTCOME).

Patients with low-moderate LDL-C levels had a higher plaque burden as expressed by the total number of segments with any plaque, non-calcified/mixed or calcified plaques. Non-calcified/mixed plaques were particularly overrepresented in the lowest LDL-C group (LDL-C < 70 mg/dL).

To control for potential bias from the inclusion of patients with prior elective revascularization or MI, more represented in the lower LDL-C groups, a sensitivity analysis was performed in the subgroup of 356 patients without any previous history of IHD events (see Supplementary data online, *Table S2*). In this subpopulation, patients in the lower LDL-C groups still demonstrated a significantly higher prevalence of more severe/extensive CAD, a higher Leiden CAD Risk score and greater overall plaque burden, confirming the consistency of the main findings.

### Clinical predictors of CAD severity/extent and CAD risk

The results of the multivariable logistic regression analyses to estimate the association of clinical characteristics and risk factors with Leiden score ≥ 5 (Primary OUTCOME) are reported for the whole population in *Table 3*.

**Table 3:** Multivariable analysis of the association between clinical characteristics, risk factors, and primary OUTCOME in the whole population

	Model 1		Model 1A		Model 2		Model 2A	
	OR (95% CI)	*P* value	OR (95% CI)	*P* value	OR (95% CI)	*P* value	OR (95% CI)	*P* value
Age	**1.10 (1.07–1.13)**	**<0.001**	**1.09 (1.06–1.11)**	**<0.001**	**1.09** (**1.06–1.11)**	**<0**.**001**	**1.08** (**1.05–1.11)**	**<0**.**001**
Sex male	**2.91 (1.80–4.72)**	**<0.001**	**2.90 (1.76–4.76)**	**<0.001**	**2.87** (**1.75–4.71)**	**<0**.**001**	**2.69** (**1.62–4.47)**	**<0**.**001**
Smoking	1.57 (0.91–2.69)	0.105	1.60 (0.92–2.79)	0.100	1.59 (0.91–2.78)	0.100	1.64 (0.92–2.89)	0.092
Family History CAD	1.20 (0.78–1.85)	0.404	1.16 (0.74–1.82)	0.525	1.19 (0.76–1.87)	0.441	1.14 (0.72–1.81)	0.582
LDL-C ≥ 130 mg/dL	1.61 (0.89–2.91)	0.113	**1.85 (1.00–3.40)**	**0.049**	1.48 (0.81–2.71)	0.202	1.64 (0.88–3.05)	0.118
LDL-C 100–129 mg/dL	REF		REF		REF		REF	
LDL-C 70–99 mg/dL	**2.15 (1.23–3.76)**	**0.008**	1.74 (0.96–3.14)	0.067	**1.97** (**1.11–3.52)**	**0**.**021**	1.74 (0.95–3.20)	0.074
LDL-C < 70 mg/dL	**2.92 (1.54–5.34)**	**0.001**	**2.36 (1.19–4.69)**	**0.014**	**2.42** (**1.24–4.72)**	**0**.**001**	**2.12** (**1.05–4.30)**	**0**.**037**
MeS	**2.51 (1.54–4.08)**	**<0.001**	**2.12 (1.26–3.56)**	**0.004**				
Hypertension					1.34 (0.79–2.28)	0.279	1.01 (0.53–1.92)	0.983
Pre-diabetes					**1.86** (**1.11–3.09)**	**0**.**018**	**1.90** (**1.12–3.21)**	**0**.**017**
Diabetes					**4.18** (**2.06–8.50)**	**<0**.**001**	**6.13** (**1.93–19.45)**	**0**.**002**
High BMI					1.35 (0.78–2.37)	0.287	1.17 (0.66–2.09)	0.600
Low HDL-C					0.82 (0.47–1.41)	0.462	0.88 (0.50–1.53)	0.639
High TG					**1.75** (**1.02–3.01)**	**0**.**042**	1.69 (0.96–2.95)	0.068

*P* values <0.05 (bold) show statistically significant differences. Model 1A and 2A are adjusted for all medications. CAD, coronary artery disease; LDL-C, LDL-cholesterol; MeS, metabolic syndrome; BMI, body mass index; HDL-C, HDL-cholesterol; TG: triglycerides. See Methods for definition of each component of MeS.

Age, male sex, the lowest LDL-C (< 70 mg/dL) category and MeS were independently associated with the OUTCOME without and with adjustment for all medications assumed (*Table 3*, Models 1 and 1A). When MeS was substituted with single cardiometabolic risk components, age, male sex, the lowest LDL-C category, and pre-diabetes or diabetes remained independently associated with the OUTCOME without and with adjustment for medications (*Table 3*, Models 2 and 2A). In addition, the sensitivity analysis performed in the subgroup of patients not treated with statins confirmed the main results with the lowest LDL-C category (OR 5.44, 95% CI: 1.59–18.65) and MeS (OR 2.60, 95% CI: 1.12–6.02) or the lowest LDL-C category (OR 4.33, 95% CI: 1.21–15.50) and diabetes (OR 5.84, 95% CI: 1.65–20.72) being independently associated with the OUTCOME together with age and male sex.

When the Secondary OUTCOME was considered, age, male sex, the lowest LDL-C category and Mes were independently associated with more severe and/or extensive CAD, without and with adjustment for medications assumed. Among single MeS components, only pre-diabetes remained independently associated with the Secondary OUTCOME after adjustment for medications (see Supplementary data online, *Table S3*). Results were superimposable in the sensitivity analysis in patients without prior IHD events (see Supplementary data online, *Table S4*).

**Figure 2:**
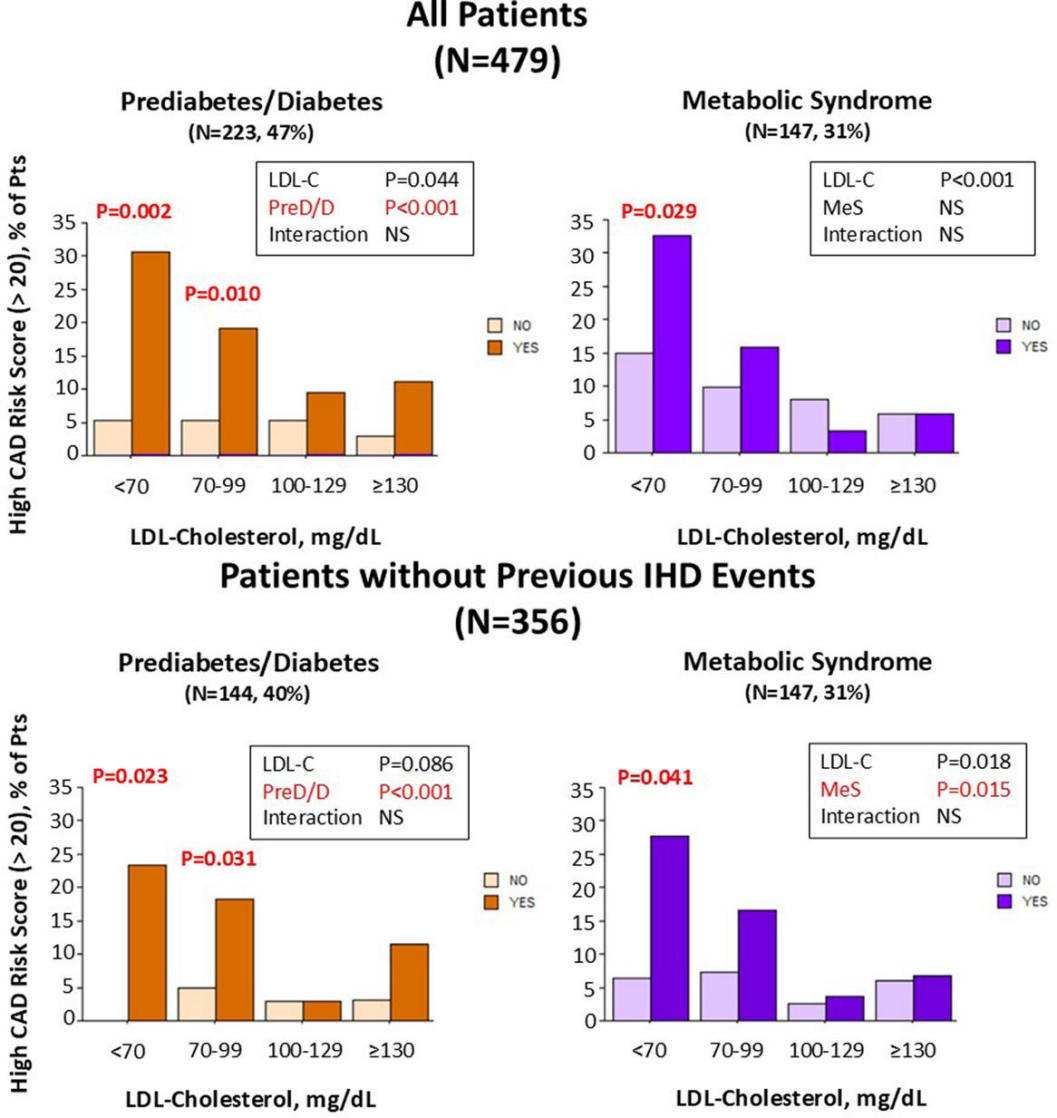
Results of ANOVA test for interaction between MeS or pre-diabetes-diabetes and LDL-C levels on frequency of high CAD risk score. Bar graphs representing the frequency (%) of the high Leiden CAD Risk score (>20) in each LDL-C group according to absence (light colours) or presence (dark colours) of MeS or pre-diabetes/diabetes in the whole population (upper panel) or the subpopulation without IHD events (lower panel). *P* values from the ANOVA table are reported in the upper right position of each graph; *P* values for comparison in the same LDL-C group are reported when significant.

The possible interacting effects of MeS or pre-diabetes-diabetes and the categories of LDL-C on the frequency of high CAD risk score (Leiden score > 20) were assessed in the whole population (*Figure 2* upper panel). Categories of LDL-C (decreasing levels) and the presence of diabetes/pre-diabetes were significantly associated with higher frequency of high CAD risk score without significant overall interaction. Nonetheless, patients with pre-diabetes/diabetes or MeS, as compared with patients without, had a significantly higher frequency of high CAD risk score in the group with LDL-C < 70 mg/dL (31% vs. 5%, *P* = 0.002 and 33% vs. 15%, *P* = 0.029, respectively). Patients with pre-diabetes/diabetes had also a significantly higher frequency of high CAD risk score in the group with LDL-C 70–99 mg/dL (19% vs. 5%, *P* = 0.010). In the subpopulation of patients without previous IHD events interaction patterns were similar (*Figure 2* lower panel).

### Effects of the interaction between biochemical indices of cardiometabolic risk and LDL-C levels on CAD risk score and plaque burden

**Figure 3:**
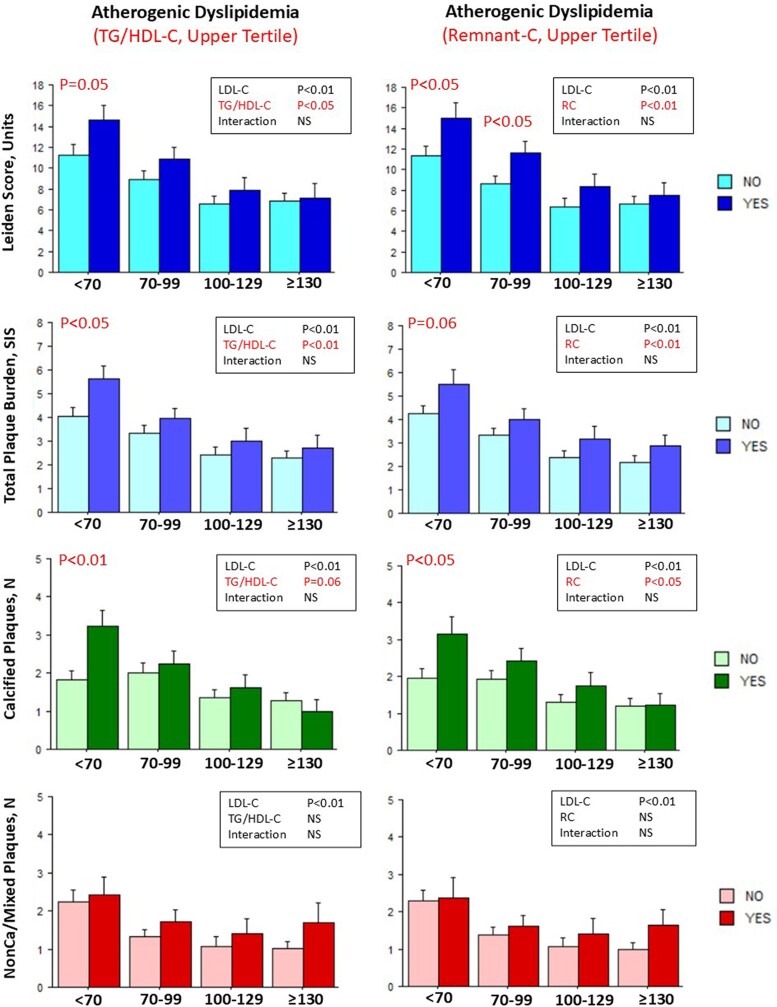
Results of ANOVA test for interaction between indices of atherogenic dyslipidemia and LDL-C levels on CAD risk score and plaque burden. Bar graphs representing values (mean ± SE) of the Leiden score, total plaque burden (SIS), calcified plaque number, and non-calcified/mixed plaque number in each LDL-C group according to absence (light colours) or presence (dark colours) of upper tertile of biochemical indices of atherogenic dyslipidemia. *P* values from the ANOVA table are reported in the upper right position of each graph and *P* values for comparison in the same LDL-C group are reported when significant. TG/HDL-C, triglycerides/high density lipoprotein cholesterol ratio; remnant-C, remnant cholesterol; see Methods section for cut-off values identifying the upper tertile of each biochemical index of cardiometabolic risk.

**Figure 4:**
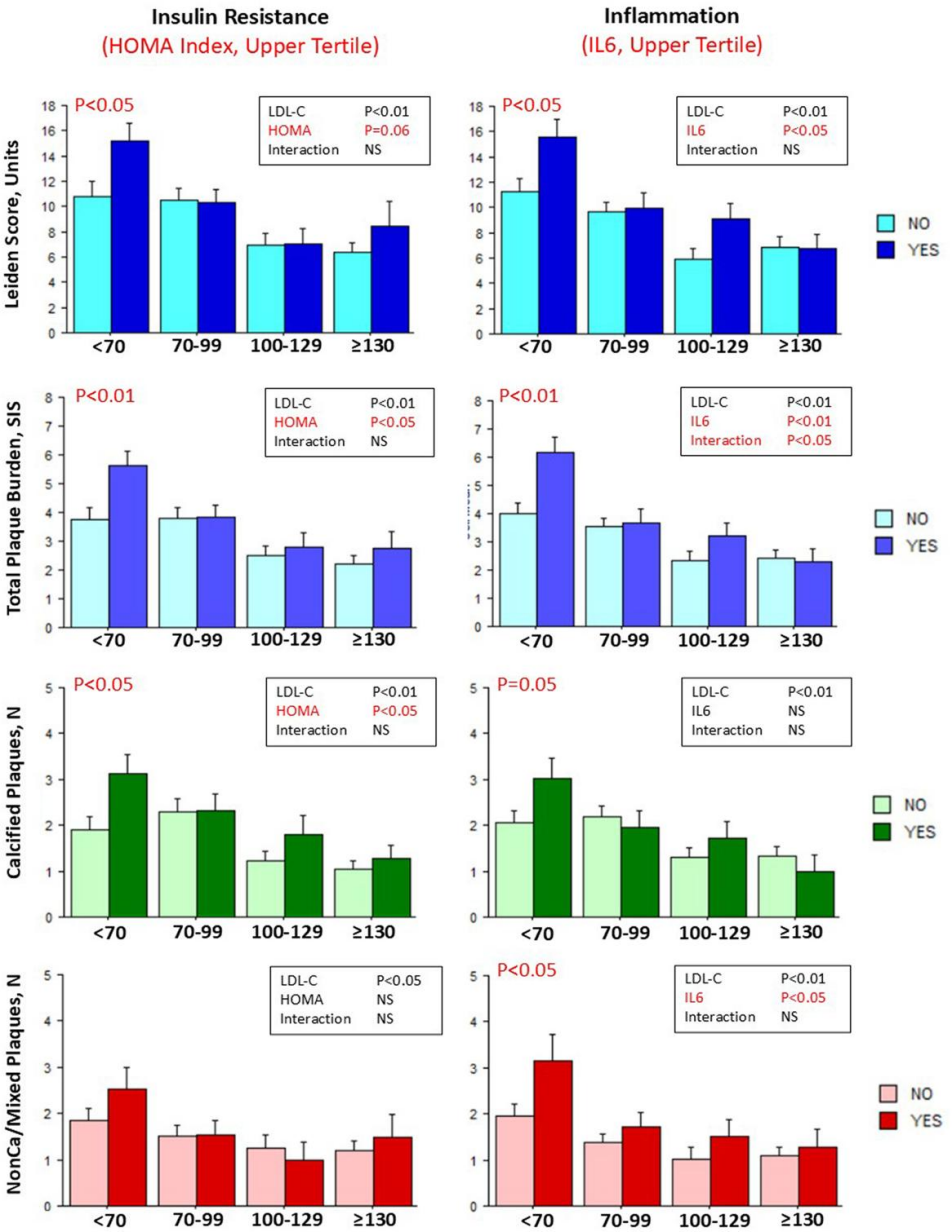
Results of ANOVA test for interaction between indices of insulin resistance, low-grade systemic inflammation and LDL-C levels on CAD risk score and plaque burden. Bar graphs representing values (mean ± SE) of the Leiden score, total plaque burdenSIS, calcified plaque number and non-calcified/mixed plaque number in each LDL-C group according to absence (light colours) or presence (dark colours) of upper tertile of biochemical indices of insulin resistance and low-grade systemic inflammation. *P* values from the ANOVA table are reported in the upper right position of each graph and *P* values for comparison in the same LDL-C group are reported when significant. HOMA index, homeostatic model assessment index; IL6, interleukin 6. See Methods section for cut-off values identifying the upper tertile of each biochemical index of cardiometabolic risk.

The possible interacting effects of biochemical indices of cardiometabolic risk and the categories of LDL-C on CAD risk score (continuous values of Leiden score) and plaque burden, defined by number of segments with any plaque (SIS), non-calcified/mixed or calcified plaques, were assessed in the whole population. Categories of LDL-C and indices of atherogenic dyslipidaemia (upper tertile of TG/HDL-C and remnant-C) were significantly associated with higher Leiden score, total and calcified plaque burden (*Figure 3*). Whereas overall interaction was not significant, patients with atherogenic dyslipidaemia, as compared with patients without, had higher Leiden score, total and calcified plaque burden in the group with LDL-C < 70 mg/dL (and also in the group with LDL-C 70–99 mg/dL for remnant-C and Leiden score). A similar pattern was observed for the association of categories of LDL-C and insulin resistance (upper tertile of the HOMA index) with higher Leiden score, total and calcified plaque burden in patients with insulin resistance and LDL-C < 70 mg/dL (*Figure 4*, left panels). On the other hand, the index of low-grade systemic inflammation (upper tertile of IL6) was also significantly associated with non-calcified/mixed plaque burden in particular in the lowest LDL-C group (*Figure 4*, right panels).

In patients without prior IHD events (see Supplementary data online, Figure Supplementary Material) the association patterns across LDL-C categories were similar for the index of insulin resistance and less evident for indices of atherogenic dyslipidaemia. Conversely, the interaction between low-grade systemic inflammation and LDL-C categories was more evident and significant for all CCTA-derived measurements. Patients with both elevated IL6 levels and LDL-C < 70 mg/dL had the highest Leiden risk score and the highest plaque burden (including either calcified and non-calcified/mixed plaques).

## Discussion

In this cross-sectional study of patients with CCS, undergoing high-quality CCTA and a comprehensive clinical and biochemical characterization, cardiometabolic risk—defined by the presence of MeS, diabetes or pre-diabetes and associated biochemical profiles—was more frequent in subjects with low LDL-C. Moreover, cardiometabolic risk was associated with CCTA-derived clinical indices of severe/extensive and high-risk CAD particularly in patients with low LDL-C. Noteworthy, this association was independent of traditional risk factors, ongoing medical therapy, and was confirmed in subjects without a prior history of IHD events.

Over the past decades, MeS has received particular clinical attention due to its high and rising prevalence, paralleling that of Type-2 diabetes.^18–22^ Both MeS and Type 2 diabetes or pre-diabetes are well-established predictors of cardiovascular and all-cause mortality.^1,12,13,33^ However, their specific association with CCTA-derived indices of CAD severity/extension and risk in patients with CCS has been investigated only recently. Analyses from the PARADIGM study demonstrated that established Type-2 diabetes, pre-diabetes, or the triglyceride-glucose index, a surrogate marker of insulin resistance, were independent predictors of adverse plaque features, coronary plaque progression, and poorer prognosis,^34,35^ even after adjustment for statin use^36^ as well as in patients without baseline coronary disease.^37^ Interestingly, in a recent post-hoc analysis of trials involving serial coronary intravascular ultrasonography measurements in patients with or without diabetes, higher (on-treatment) HbA1c levels were associated with coronary plaque progression and major adverse cardiovascular events (MACE) independently of other achieved risk factors control.^38^ The findings of our study extend recent evidence of the role of cardiometabolic risk in patients with CCS in whom we observed a higher prevalence of MeS, and particularly diabetes and pre-diabetes, at lower LDL-C levels and the association of this combined cardiometabolic condition with higher CAD severity/extent and risk, independently of other risk factors and medical treatments.

While increasingly lower LDL-C levels under treatment are convincingly associated with better outcome in multiple trials, there is also evidence that either genetically determined low LDL-C^23^ as well as intensive statin treatment^39–42^ may be associated with impaired glucose tolerance and higher prevalence of new-onset diabetes even if the risk/benefit ratio in terms of MACE remains in favour of low LDL-C.^23,41^

In a wide population-based study, a strong inverse association between LDL-C and incident diabetes was also recently demonstrated in a cohort of participants free of Type 2 diabetes and CVD who were followed for a median of 6 years.^43^ The increased risk of new-onset diabetes at lower LDL-C levels was documented either in statin users and non-users (48% of subjects) suggesting that the association between low LDL-C and development of diabetes may be independent of treatment. The pathophysiologic mechanisms by which low LDL-C may increase the risk of diabetes is unclear. Genetically determined and/or pharmacologically induced low plasma LDL-C levels, may be associated with an increased density of LDL receptors with potential effects on pancreatic ß-cells function^23,41,43,44^ which may also be influenced by other mechanisms related to cholesterol metabolism.^44^ On the other hand, persons with familial hypercholesterolaemia, due to genetic defects in LDL receptor function, appear to have a lower prevalence of diabetes than unaffected relatives.^45^

The possible interaction between LDL-C levels, statin treatment, incident diabetes, and residual CAD risk in patients with CCS is complex and had not been fully investigated. In a large population of patients with suspected CAD undergoing CCTA^46^ and with a wide range of LDL-C levels the absence of coronary plaques was observed in similar proportions and was associated with similar low risk of events across all LDL-C levels. On the other hand, a high plaque burden (CAC score > 100) was associated with higher event rates in all groups but tended to be more prevalent and associated with higher risk in patients with lower LDL-C (<77 mg/dL) who also had a clearly higher prevalence of diabetes.

Our study should be considered exploratory and hypothesis generating. Nevertheless, the present results add on existing evidence, confirming the association between exposure to lower LDL-C levels and higher prevalence of diabetes or pre-diabetes in patients with CCS in whom this combined metabolic condition seems a relevant determinant of residual CAD risk. Due to the observational cross-sectional design, the clinical and treatment heterogeneity and the relatively small sample size of the population, possible temporal and causal relationships cannot be ascertained and ‘reverse causality’ cannot be excluded. Nonetheless, strengths of our study were the extensive characterization of all patients and the use of multiple parameters to evaluate plaque burden, plaque components and the CAD risk phenotype from CCTA. The association of lower LDL-C levels, diabetes and pre-diabetes and indexes of higher plaque burden was consistent using all CCTA-derived parameters including both calcified and non-calcified plaques number, stenosis severity as well as the integrated Leiden risk score. This association was confirmed either in the whole population after adjustment for all medication use or in particular in subgroups of patients not taking statins or without any previous IHD event, under a lower intensity treatment.

Patients with Type 2 diabetes mellitus (T2DM), almost 8% of the global adult population,^47^ have a 2 to 5 times higher risk of developing macrovascular disease than non-diabetic individuals as glycaemia is a persistent contributing factor for the development of vascular damage with no discernible threshold.^48^ Besides T2DM, there are a number of emerging cardiometabolic conditions, often clustering together, which are associated with increased cardiovascular diseases and in particular CAD risk.^49^ They include impaired glucose tolerance and insulin resistance (often preceding overt diabetes stages), atherogenic dyslipidaemia (characterized by increased triglycerides, lower HDL-C, and increased remnant-C) and abnormal adipose tissue function often associated with low-grade systemic inflammation.^14–17^ These conditions may contribute to a more extensive and severe coronary atherosclerosis, independently of LDL-C levels, by multiple mechanisms.^49^ Impaired glycaemic control is accompanied by increased hepatic TG synthesis, lower and dysfunctioning circulating HDL-C particles, with reduced reverse cholesterol transport properties, and high amounts of small LDL-C particles involved in the development of atherosclerosis. Moreover, impaired glucose metabolism is associated with endothelial dysfunction, oxidative stress, and low-grade systemic inflammation also fuelled by inflammatory mediators produced by dysfunctioning adipose tissue. Endothelial dysfunction promotes infiltration of inflammatory cells and the development of plaque in the artery wall. In our population higher levels of biochemical indices of atherogenic dyslipidaemia (TG/HDL-C ratio or remnant cholesterol) were associated with a significant increase in total/calcified coronary plaque burden and estimated risk in patients with the lowest LDL-C levels (*Figure 3*). A similar association at the lowest LDL-C levels was also observed for indexes of insulin resistance (HOMA index) while low-grade systemic inflammation (IL6) was also associated with non-calcified/mixed (less stabilized) plaque burden (*Figure 4*). Similar results were obtained in patients without previous IHD events (under a lower treatment intensity) in whom the interaction between high IL6 and low LDL-C on all types of plaques and on the Leiden risk score was even more evident (see Supplementary data online, Figure Supplementary material). The present results are consistent with those recently reported in a large population of patients with established coronary disease, under statin treatment, undergoing coronary revascularization, in which patients with both higher C-reactive protein and low LDL-C had the highest risk of major cardiovascular events at follow-up even if, after adjusting for other risk factors including diabetes, systemic inflammation remained the major predictor of risk.^50^ Further studies are needed to investigate the complex pathogenetic relationships between LDL-C levels, other glucose and lipids metabolic abnormalities and low-grade systemic inflammation as determinants of residual CAD risk.

## Limitations

Our study has several limitations. The relatively small sample size, due to the extensive clinical and biochemical characterization required, as well as the lack of information on statin dose, precluded detailed subgroup analyses (e.g. sex, age, high-intensity vs. low-intensity statin treatment or alternative LDL-C lowering strategies, presence vs. absence of pre-diabetes-diabetes, etc.) that could have better elucidated the interpretation of our findings. The oral glucose tolerance test is recognized as the most sensitive measure of pre-diabetes and unrecognized diabetes^51–52^ thus using FPG levels in patients without known diabetes could have introduced some misclassification. Nevertheless, the clustering of high FPG with other components of the MeS could have improved the diagnostic accuracy.^52^ Moreover, due to the cross-sectional design, data on previous and evolving biochemical profiles, treatments, and exposure duration (in particular to statins) are not available, limiting inference on temporal or causal relationship between LDL-C lowering, glucose metabolism alterations, and CAD progression. The ongoing prospective HURRICANE study may partially overcome these limitations, though the sample size was designed for proof-of concept rather than outcome validation.^24^

## Conclusions

The results of this cross-sectional study suggest that dysregulation of glucose metabolism, associated cardiometabolic profiles, and low-grade systemic inflammation, are relevant components of residual coronary atherosclerotic risk in patients with CCS and low LDL-C under current treatment.

While our efforts are currently directed to aggressively reduce LDL-C to reduce CV events, in many patients who achieved low LDL-C levels, cardiometabolic risk remains underestimated and undertreated.^22,49^ Anti-inflammatory therapies^53^ and modern glucose-lowering agents^54–55^ offer promise even if the adequate strategies to reduce residual risk in patients with stable CAD and adverse cardiometabolic profile are still under debate.^51,56^

Large, prospective, multicentre studies collecting extensive imaging, biochemical, and omics data are needed to clarify the complex relationship between lipid metabolism, glucose metabolism, and low-grade systemic inflammation in patients with CCS and to identify potential novel personalized prevention and treatment strategies to reduce residual CAD risk.

## Lead author biography


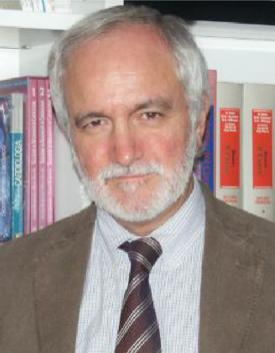


Dr. Neglia is cardiologist and nuclear medicine specialist. He is senior staff cardiologist at Fondazione Toscana Gabriele Monasterio in Pisa (FTGM) since 1997 being appointed as Head of Positron Emission Tomography till 2012 and later on as Director of Multimodality Cardiovascular Imaging Program. He is Affiliate Researcher at Institute of Clinical Physiology of the CNR in Pisa. He is also Affiliate Researcher at Institute of Life Sciences and Faculty Member of the PhD Program in Translational Medicine of Scuola Superiore Sant'Anna in Pisa.

## Supplementary Material

qyag021_Supplementary_Data

## Data Availability

The data underlying this article cannot be shared publicly due to the privacy of individuals that participated in the study. The data will be shared on reasonable request to the corresponding author.
