# Supplementary material for: Residual coronary atherosclerotic risk and low LDL-cholesterol in chronic coronary syndromes
Source: Eur Heart J Imaging Methods Pract. 2026 Feb 4;4(1):qyag021. doi: 10.1093/ehjimp/qyag021 (PMC12933505; doi:10.1093/ehjimp/qyag021)
Supplement: qyag021_Supplementary_Data [file qyag021_supplementary_data.docx]

**Table 1 Supplement. Eligibility criteria**

|  | |
| --- | --- |
| **Major Inclusion Criteria** | **Major Exclusion Criteria** |
| Patients with known or suspected stable CAD who underwent CCT for the registered studies | Overt heart failure (NYHA Class III–IV) and/or reduced systolic LV function (LVEF < 40%) |
| ≥45 and ≤75 years of age | Relevant comorbid conditions limiting expected survival to <1 year |
| Fully accessible CCT image files | CCT exam of sub-optimal quality |
| Written informed consent | No written informed consent |
| **Additional Inclusion Criteria** | **Additional Exclusion Criteria** |
| Available information on demographic data, cardiovascular risk factors, family history, history of previous IHD events, symptoms, and medications | Incomplete information |
| Available laboratory characterization including conventional circulating biomarkers of lipid and glucose metabolism, additional biomarkers of systemic inflammation, adipocytes and kidney function | Incomplete biohumoral data |

**Table 1 Supplement Legend:** CAD, coronary artery disease; CCT, cardiac computed tomography; LVEF, left ventricle ejection fraction; NYHA, New York Heart Association; IHD, Ischemic Heart Disease.

**Table 2 Supplement. CCTA-derived CAD severity/extent, CAD risk and coronary plaque burden in the subpopulation of 356 patients without previous IHD events**

| **Characteristics** | **ALL** | **LDL Cholesterol** | | | | | **LDL Cholesterol** | | |
| --- | --- | --- | --- | --- | --- | --- | --- | --- | --- |
|  | **N=357** | **Low**  **< 70 mg/dL**  **N=49** | **Moderate**  **70-99.9 mg/dL**  **N=105** | **Intermediate**  **100-129.9 mg/dL**  **N=104** | **High**  **≥ 130 mg/dL**  **N=99** | ***P Value*** | **Low-Moderate**  **< 100 mg/dL**  **N=154** | **Intermediate-High**  **≥ 100 mg/dL**  **N=203** | ***P Value*** |
| **Presence/Severity of CAD**  **(CAD-RADS2 Score)** |  | | | | | | | | |
| Absent CAD (0) | 107 (30) | 7 (14) | 27 (26) | 42 (40) | 31 (31) | *<0.01* | 34 (22) | 73 (36) | *<0.005* |
| Non Obstructive CAD (1-2) | 185 (52) | 32 (65) | 51 (48) | 50 (48) | 52 (53) |  | 83 (54) | 102 (50) |  |
| Obstructive CAD (3-4-5) | 65 (18) | 10 (21) | 27 (26) | 12 (12) | 16 (16) |  | 37 (24) | 28 (14) |  |
| **Extension of CAD**  **(CAD-RADS2 P Score)** |  |  |  |  |  |  |  |  |  |
| Absent CAD (0) | 107 (30) | 7 (14) | 27 (26) | 42 (40) | 31 (31) | *<0.001* | 34 (22) | 73 (36) | *<0.001* |
| Less Extensive (1-2) | 153 (43) | 17 (35) | 43 (41) | 42 (40) | 51 (52) |  | 60 (39) | 93 (46) |  |
| More Extensive (3-4) | 97 (27) | 25 (51) | 35 (33) | 20 (20) | 17 (17) |  | 60 (39) | 37 (18) |  |
| **Secondary OUTCOME** |  |  |  |  |  |  |  |  |  |
| Obstructive and/or more extensive CAD | 116 (32) | 25 (51) | 43 (41) | 24 (23) | 24 (24) | *<0.001* | 68 (44) | 48 (24) | *<0.001* |
| **CAD Risk (Leiden Score)** |  |  |  |  |  |  |  |  |  |
| Score (Continuous) | 7.5±7.3 | 10.5±7.5 **^,##^ | 8.6±7.8 ** | 5.8±6.2 | 6.6±7.0 | *<0.001* | 9.2±7.8 | 6.2±6.6 | *<0.001* |
| High Risk (Score > 20) | 27 (8) | 7 (14) | 11 (10) | 3 (3) | 6 (6) | ***<0.05*** | 18 (12) | 9 (4) | ***<0.05*** |
| **Primary OUTCOME** |  |  |  |  |  |  |  |  |  |
| Moderate-High Risk (Score ≥ 5) | 190 (53) | 33 (67) | 64 (61) | 45 (43) | 48 (48) | *<0.01* | 97 (63) | 93 (46) | *<0.005* |
| **Plaque Burden and Type** |  |  |  |  |  |  |  |  |  |
| Segment Involvement Score (SIS) | 2.8±3.0 | 4.4±3.5 ^†,^**^,##^ | 3.3±3.3 **^,#^ | 2.1±2.6 | 2.3±2.6 | *<0.001* | 3.7±3.4 | 2.2±2.6 | *<0.001* |
| Non-Calcified/Mixed Plaques (N) | 1.2±2.1 | 2.0±2.9 ^†,^**^,##^ | 1.3±2.0 | 0.8±1.7 | 1.1±1.9 | *<0.005* | 1.5±2.3 | 0.9±1.8 | *<0.01* |
| Calcified Plaques (N) | 1.6±2.2 | 2.3±2.6 *^,##^ | 2.0±2.6 *^,##^ | 1.4±2.0 | 1.2±1.8 | *<0.005* | 2.1±2.5 | 1.3±1.9 | *<0.001* |

**Table 2 Supplement Legend:** Continuous variables are presented as mean ± standard deviation and logarithmic transformed for comparison when necessary. Categorical variables are presented as absolute N and (%). *P values* <0.05 indicate statistically significant differences among the 4 groups. † = P<0.05 vs Moderate LDL-C group. *^,^** = P<0.05, P<0.01 vs Intermediate LDL-C group. ^#,##^ = P<0.05, P<0.01 vs High LDL-C group. CAD: Coronary Artery Disease; CAD-RADS2 Scores: see Methods’ section for details; OUTCOMEs: see also Methods’ section for details.

**Table 3 Supplement. Multivariable analysis of the association between clinical characteristics, risk factors and Secondary OUTCOME in the whole population**

|  | ***Model 1*** | | ***Model 1A*** | | ***Model 2*** | | ***Model 2A*** | |
| --- | --- | --- | --- | --- | --- | --- | --- | --- |
|  | ***OR (95% CI)*** | ***p***  ***Value*** | ***OR (95% CI)*** | ***p***  ***Value*** | ***OR (95% CI)*** | ***p***  ***Value*** | ***OR (95% CI)*** | ***p***  ***Value*** |
| **Age** | **1.09 (1.07-1.12)** | **<0.001** | **1.08 (1.05-1.11)** | **<0.001** | **1.09 (1.06-1.12)** | **<0.001** | **1.08 (1.05-1.11)** | **<0.001** |
| **Sex Male** | **5.30 (3.12-8.97)** | **<0.001** | **6.19 (3.48-11.01)** | **<0.001** | **5.27 (3.08-8.99)** | **<0.001** | **5.96 (3.33-10.64)** | **<0.001** |
| **Smoking** | 1.22 (0.69-2.14) | 0.496 | 1.28 (0.68-2.41) | 0.436 | 1.26 (0.71-2.26) | 0.435 | 1.31 (0.69-2.49) | 0.411 |
| **Family History CAD** | 1.38 (0.88-2.17) | 0.158 | 1.16 (0.71-1.90) | 0.561 | 1.35 (0.85-2.14) | 0.204 | 1.16 (0.70-1.91) | 0.573 |
| **LDL-C ≥ 130 mg/dL** | 1.16 (0.61-2.22) | 0.649 | 1.56 (0.78-3.14) | 0.212 | 1.18 (0.61-2.28) | 0.632 | 1.53 (0.75-3.10) | 0.243 |
| **LDL-C 100-129 mg/dL** | REF |  | REF |  | REF |  | REF |  |
| **LDL-C 70-99 mg/dL** | **2.82 (1.59-5.01)** | **<0.001** | **1.97 (1.04-3.71)** | **0.038** | 2.69 (1.49-4.87) | 0.001 | 1.91 (0.99-3.38) | 0.054 |
| **LDL-C < 70 mg/dL** | **7.25 (3.69-14.25)** | **<0.001** | **5.29 (2.48-11.29)** | **<0.001** | **6.86 (3.38-13.92)** | **<0.001** | **5.12 (2.35-11.17)** | **<0.001** |
| **MeS** | **2.77 (1.69-4.53)** | **<0.001** | **2.44 (1.42-4.22)** | **0.001** | --- | --- | --- | --- |
| **Hypertension** | --- | --- | --- | --- | 1.72 (0.97-3.06) | 0.063 | 1.18 (0.58-2.42) | 0.646 |
| **Prediabetes** | --- | --- | --- | --- | **1.95 (1.14-3.32)** | **0.014** | **2.37 (1.31-4.28)** | **0.004** |
| **Diabetes** | --- | --- | --- | --- | **2.09 (1.12-3.90)** | **0.020** | 1.63 (0.68-3.95) | 0.276 |
| **High BMI** | --- | --- | --- | --- | 1.49 (0.85-2.63) | 0.167 | 1.25 (0.67-2.32) | 0.489 |
| **Low HDL-C** | --- | --- | --- | --- | 1.36 (0.77-2.38) | 0.286 | 1.68 (0.91-3.10) | 0.097 |
| **High TG** | --- | --- | --- | --- | 1.57 (0.91-2.70) | 0.104 | 1.24 (0.68-2.25) | 0.482 |

**Table 3 Supplement Legend:** *P values* <0.05 (Bold) show statistically significant differences. Model 1A and 2A are adjusted for all medications. CAD: Coronary Artery Disease; LDL-C: Low Density Lipoprotein Cholesterol; MeS: Metabolic Syndrome; BMI: Body Mass Index; HDL-C: High Density Lipoprotein Cholesterol; TG: Triglycerides. See Methods’ section for definition of each component of MeS.

**Table 4 Supplement.** **Multivariable analysis of the association between clinical characteristics, risk factors and Secondary OUTCOME in the subpopulation of 356 patients without previous IHD events**

|  | ***Model 1*** | | ***Model 1A*** | | ***Model 2*** | | ***Model 2A*** | |
| --- | --- | --- | --- | --- | --- | --- | --- | --- |
|  | ***OR (95% CI)*** | ***p***  ***Value*** | ***OR (95% CI)*** | ***p***  ***Value*** | ***OR (95% CI)*** | ***p***  ***Value*** | ***OR (95% CI)*** | ***p***  ***Value*** |
| **Age** | **1.11 (1.08-1.15)** | **<0.001** | **1.11 (1.07-1.15)** | **<0.001** | **1.11 (1.07-1.15)** | **<0.001** | **1.11 (1.07-1.15)** | **<0.001** |
| **Sex Male** | **4.24 (2.31-7.76)** | **<0.001** | **4.84 (2.57-9.12)** | **<0.001** | **4.39 (2.36-8.17)** | **<0.001** | **4.86 (2.55-9.25)** | **<0.001** |
| **Smoking** | 1.61 (0.84-3.10) | 0.155 | 1.59 (0.80-3.14) | 0.183 | 1.78 (0.90-3.51) | 0.100 | 1.72 (0.85-3.47) | 0.131 |
| **Family History CAD** | 1.57 (0.92-2.69) | 0.098 | 1.44 (0.83-2.50) | 0.200 | 1.45 (0.83-2.53) | 0.188 | 1.37 (0.78-2.43) | 0.275 |
| **LDL-C ≥ 130 mg/dL** | 1.57 (0.75-3.27) | 0.228 | 1.76 (0.83-3.74) | 0.142 | 1.61 (0.75-3.44) | 0.223 | 1.78 (0.82-3.87) | 0.143 |
| **LDL-C 100-129 mg/dL** | REF |  | REF |  | REF |  | REF |  |
| **LDL-C 70-99 mg/dL** | **2.09 (1.06-4.14)** | **0.033** | 1.69 (0.82-3.47) | 0.152 | **2.04 (1.01-4.15)** | **0.048** | 1.72 (0.82-3.60) | 0.153 |
| **LDL-C < 70 mg/dL** | **3.46 (1.50-7.99)** | **0.004** | **2.68 (1.09-6.60)** | **0.032** | 3.13 (1.28-7.65) | 0.012 | **2.56 (1.00-6.55)** | **0.049** |
| **MeS** | **2.67 (1.51-4.73)** | **<0.001** | **2.15 (1.16-3.99)** | **0.015** | --- | --- | --- | --- |
| **Hypertension** | --- | --- | --- | --- | **2.21 (1.05-4.63)** | **0.036** | 1.94 (0.80-4.69) | 0.142 |
| **Prediabetes** | --- | --- | --- | --- | 1.76 (0.93-3.30) | 0.081 | **1.94 (1.01-3.72)** | **0.046** |
| **Diabetes** | --- | --- | --- | --- | **2.22 (1.09-4.51)** | **0.028** | 1.50 (0.58-3.87) | 0.405 |
| **High BMI** | --- | --- | --- | --- | 1.62 (0.83-3.14) | 0.157 | 1.53 (0.78-3.02) | 0.220 |
| **Low HDL-C** | --- | --- | --- | --- | 0.90 (0.45-1.80) | 0.759 | 1.03 (0.50-2.11) | 0.945 |
| **High TG** | --- | --- | --- | --- | **1.96 (1.05-3.69)** | **0.036** | 1.71 (0.89-3.30) | 0.110 |

**Table 4 Supplement Legend:** *P values* <0.05 (Bold) show statistically significant differences. Model 1A and 2A are adjusted for major risk factors modifying medications (Statins, RAS inhibitors, Beta-Blockers, Antidiabetics). CAD: Coronary Artery Disease; LDL-C: Low Density Lipoprotein Cholesterol; MeS: Metabolic Syndrome; BMI: Body Mass Index; HDL-C: High Density Lipoprotein Cholesterol; TG: Triglycerides. See Methods’ section for definition of each component of MeS


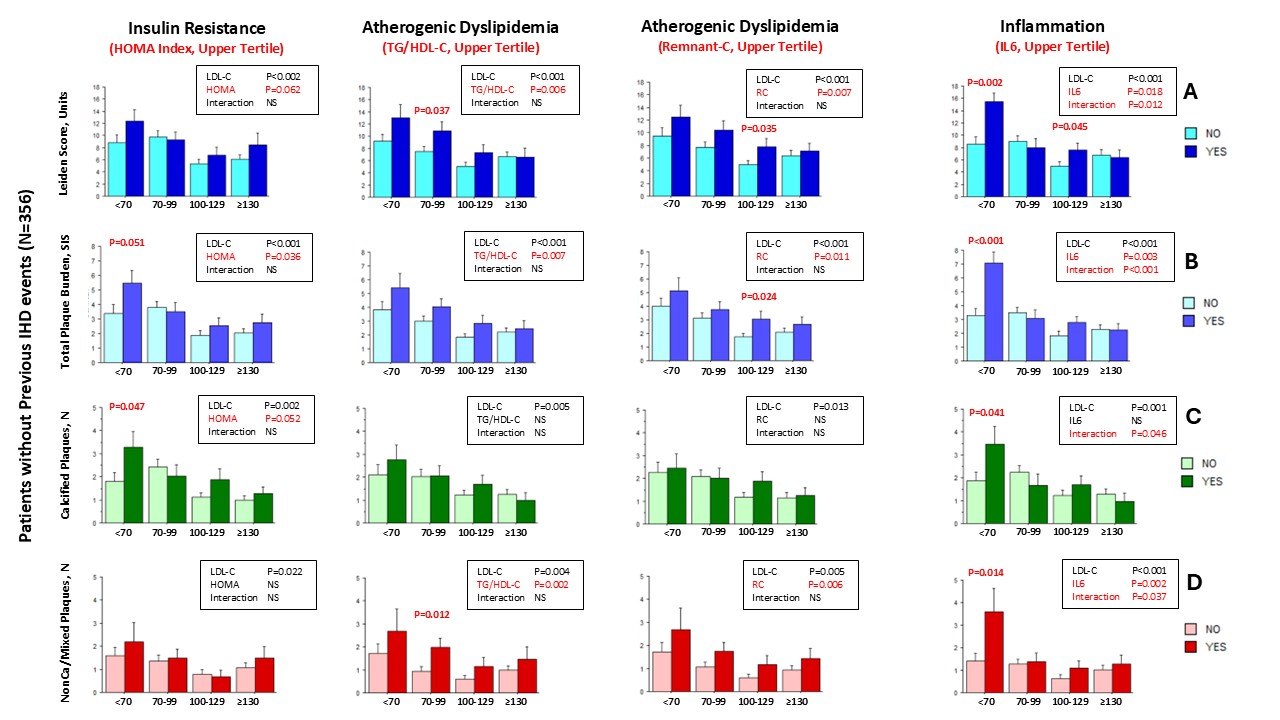


**Figure Supplement. Results of ANOVA test for interaction between biochemical indices of cardiometabolic risk and LDL-C levels on CAD risk score and plaque burden in the subpopulation without previous Ischemic Heart Disease events**.

Bar graphs representing values (mean±SE) of the Leiden Score (Panel A), Total Plaque Burden (SIS) (Panel B), Calcified Plaque Number (Panel C) and Noncalcified/Mixed Plaque Number (Panel D) in each low-density lipoprotein cholesterol (LDL-C) group according to absence (light colours) or presence (dark colours) of Upper Tertile of biochemical indices of cardiometabolic risk. P values from the ANOVA table are reported in the upper right position of each graph and P values for comparison in the same LDL-C group are reported when significant.

HOMA index=Homeostatic Model Assessment index; TG/HDL-C=Triglicerydes/ High density lipoprotein Cholesterol ratio; Remnant-C= Remanant Cholesterol; IL6=Interleukin 6. See Methos’ section for cut-off values identifying the Upper Tertile of each biochemical index of cardiometabolic risk.
